# Supplementary material for: Development of S Haplotype-Specific Markers to Identify Genotypes of Self-Incompatibility in Radish (Raphanus sativus L.)
Source: Plants (Basel). 2024 Mar 4;13(5):725. doi: 10.3390/plants13050725 (PMC10934505; doi:10.3390/plants13050725)
Supplement: Supplementary file 1 [file plants-13-00725-s001.zip › Supplementary Figure.pdf]

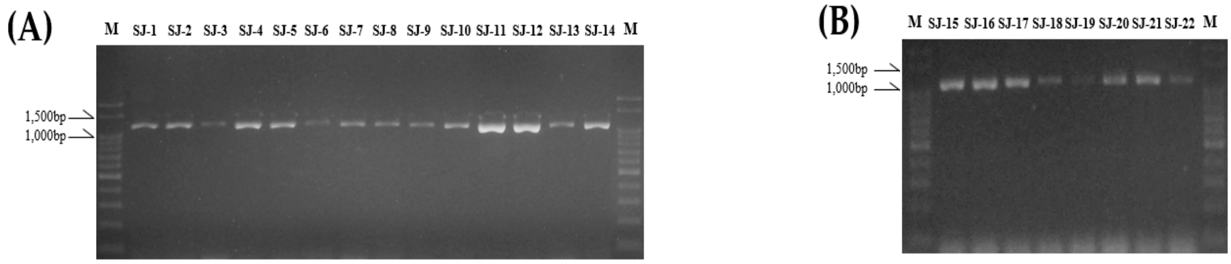

Figure S1. PCR result of 22 radish breeding lines ('SJ-1~22') using class I, II kinase domain of SRK (SRK-KD) primer set. (A) PCR amplification of class I SRK-KD using UVSRK-F + UVSRK-R primer set; (B): PCR amplification of class II SRK-KD using KS2 + KA2 primer set; M: 100bp size marker.

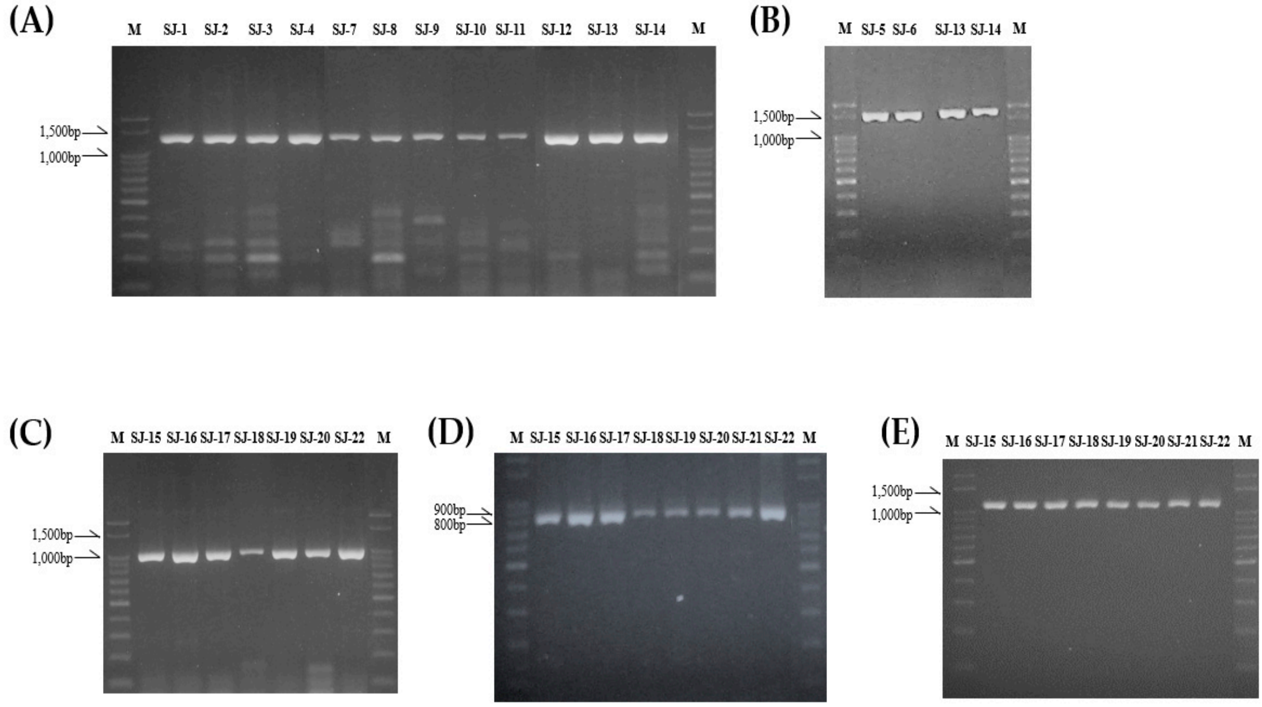

Figure S2. PCR result of 22 radish breeding lines ('SJ-1~22') using class I, II SLG primer set. (A) PCR amplification of class I SLG using SLG-I-F + SLG-I-R primer set; (B): PCR amplification of class I SLG using PS22 + SLG-I-R primer set; (C): PCR amplification of class II SLG using SLG-II-F + SLG-II-R primer set; (D): PCR amplification of class II SLG using Rs9 SLG-F + Rs9 SLG-R primer set; M: 100bp size marker; (E): PCR amplification of class II SLG using UVSLGII-F + UVSLGII-R primer set; M: 100bp sizer marker.

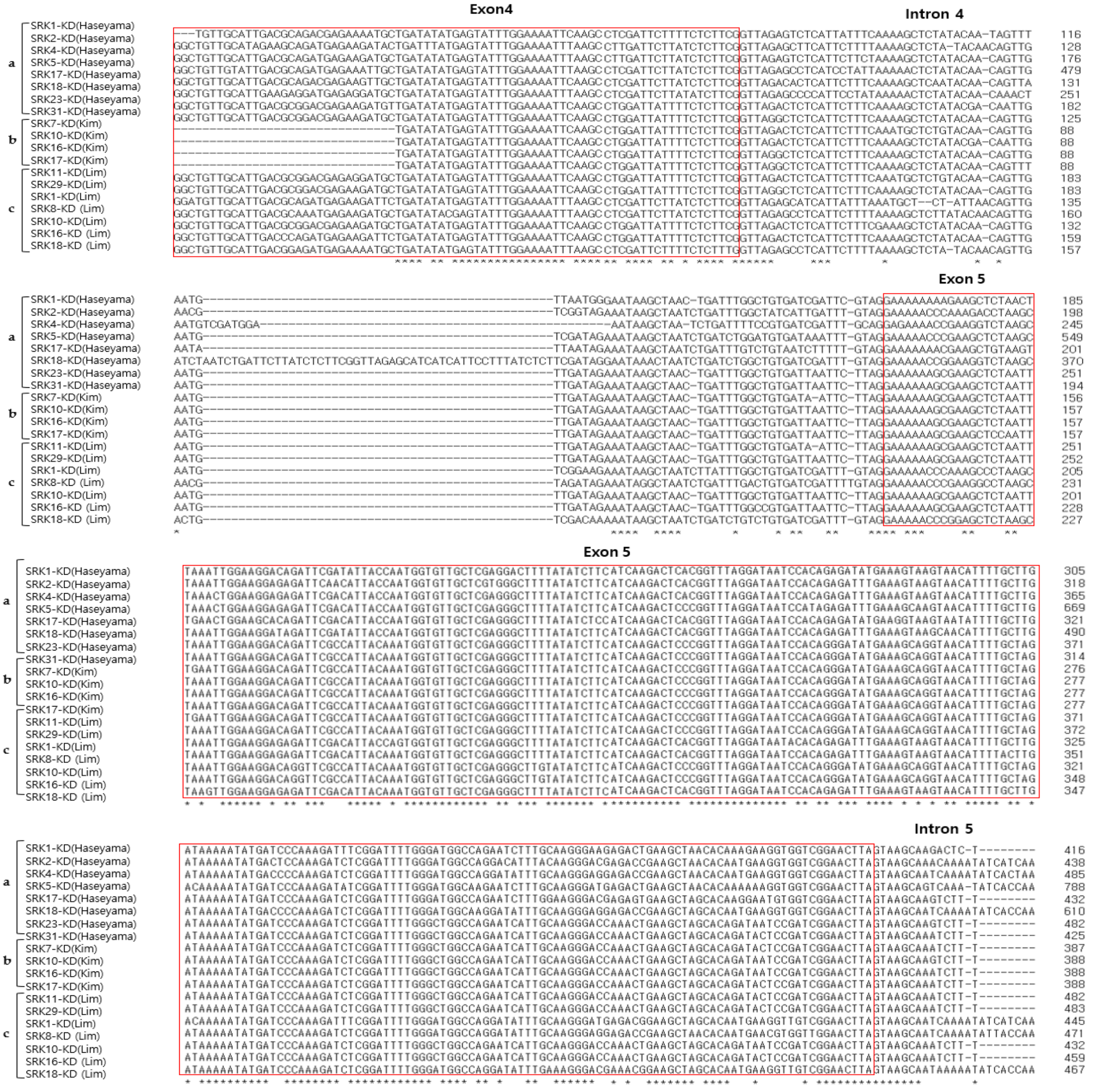

Figure S3. Multiple sequence alignment of the nucleotide sequences of the 19 class I S haplotype kinase domain of SRK alleles; a: S haplotype published by Haseyama (2018); b: S haplotype published by Kim (2016); c: S haplotype published by Lim (2002); Red box: 4th to 7th exons of the SRK; Asterisk: conserved nucleotide.

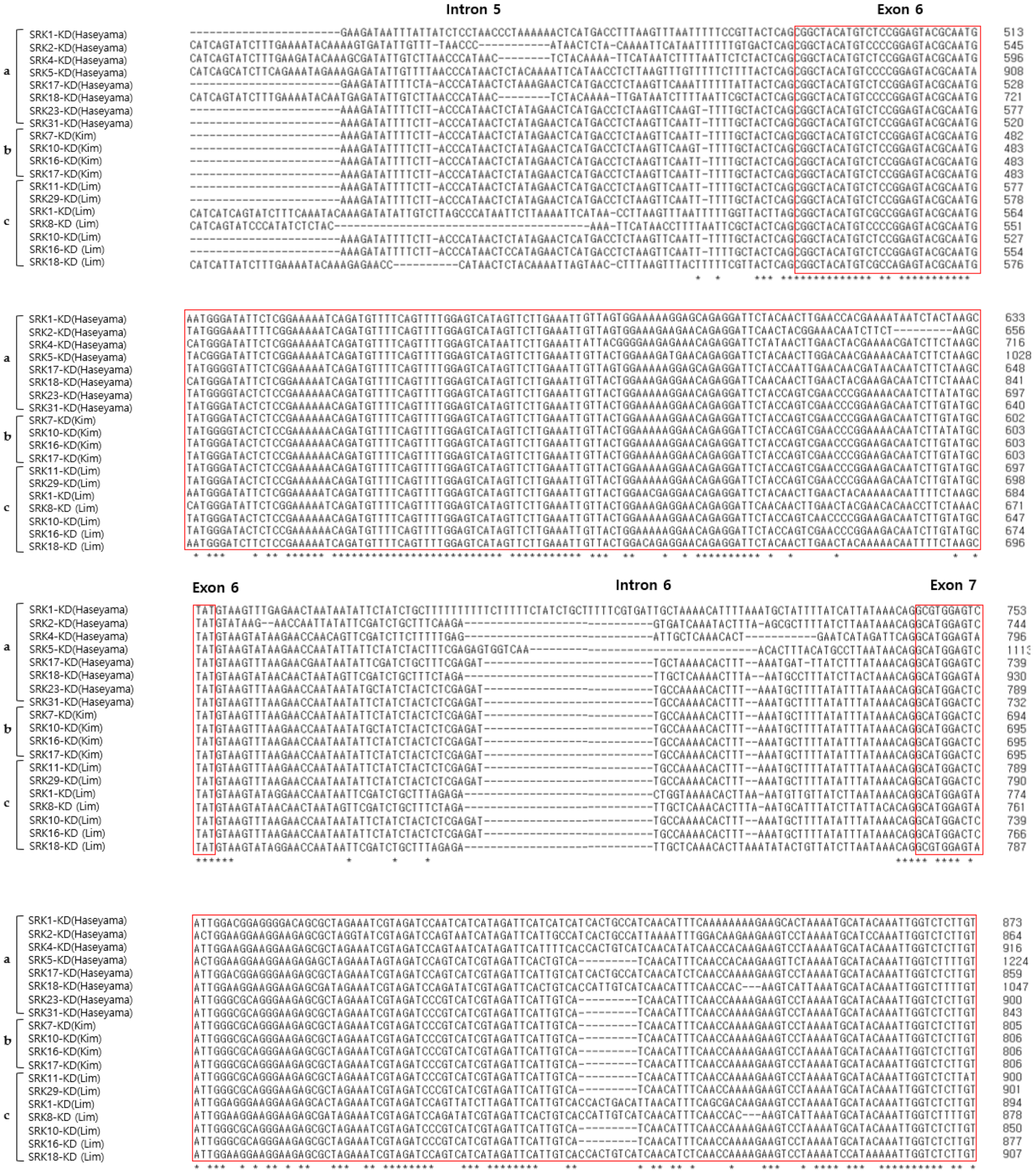

Figure S3. *Continued.*

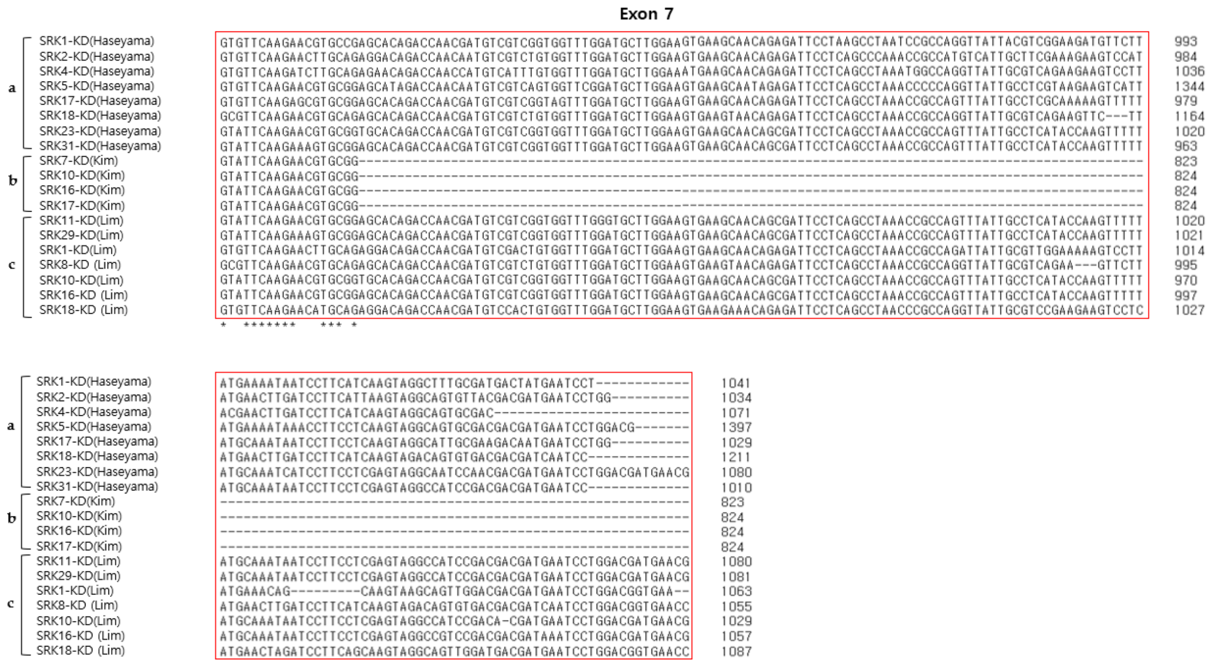

Figure S3. *Continued.*

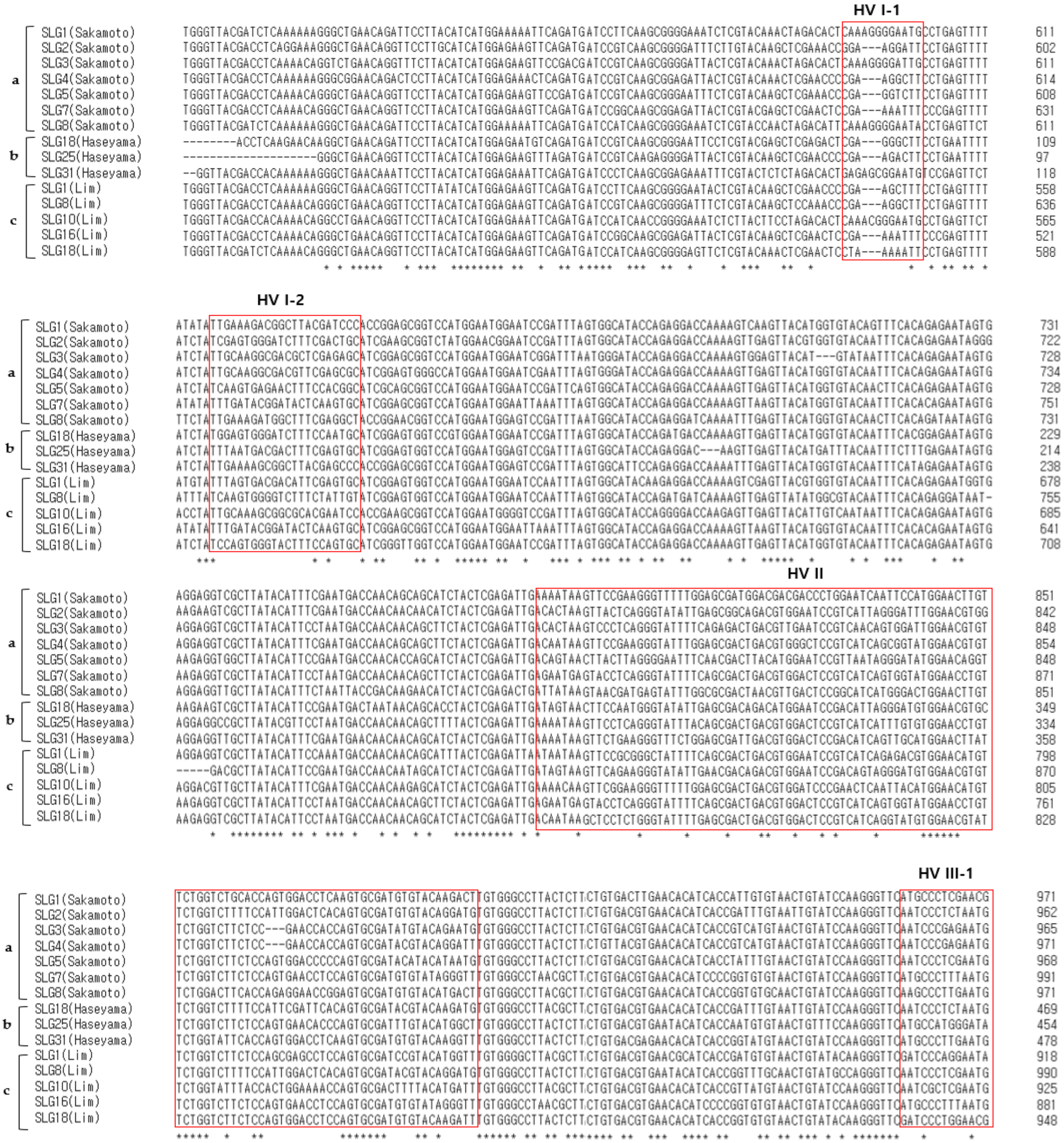

Figure S4. Multiple sequence alignment of the nucleotide sequences of the 15 class I S haplotype SLG alleles; a: S haplotype published by Sakamoto (1998); b: S haplotype published by Haseyama (2018); c: S haplotype published by Lim (2002); Red box: hypervariable region I to III of the SLG; Asterisk: conserved nucleotide.

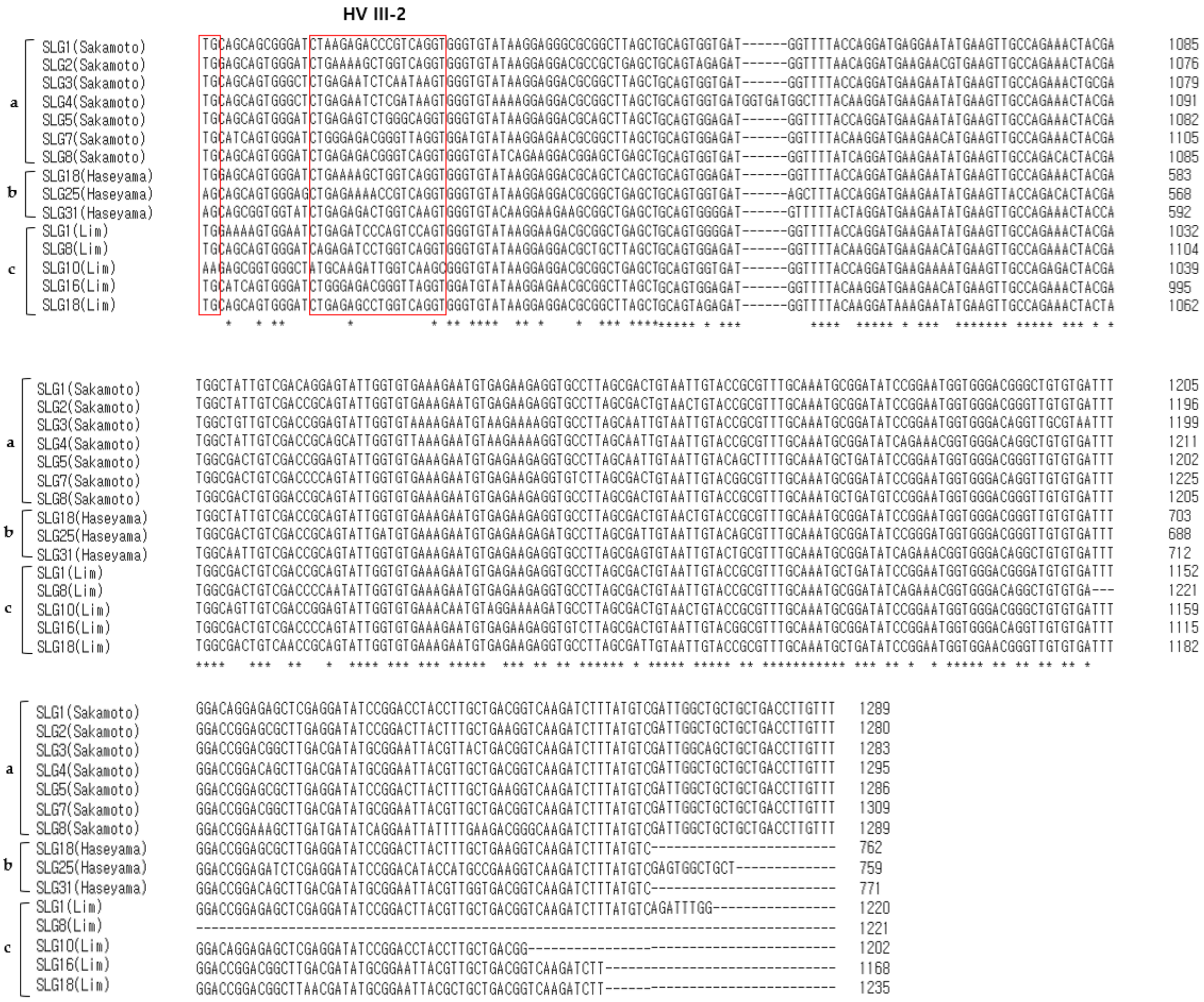

Figure S4. *Continued.*

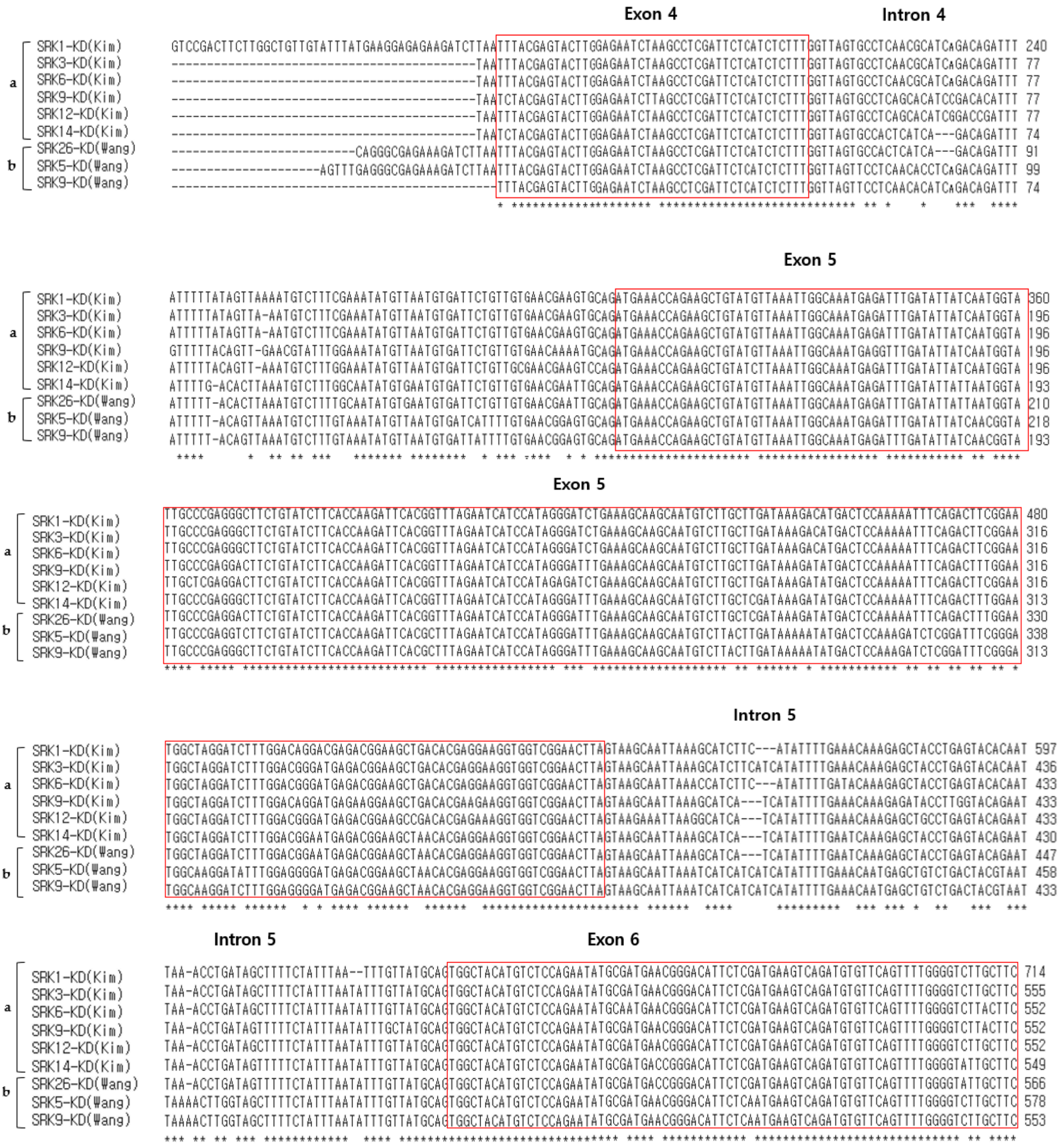

Figure S5. Multiple sequence alignment of the nucleotide sequences of the 9 class II S haplotype kinase domain of SRK alleles; a: S haplotype published by Kim (2016); b: S haplotype published by Haseyama (2018); c: S haplotype published by Lim (2002); Red box: 4th to 7th exons of the SRK; Asterisk: conserved nucleotide.

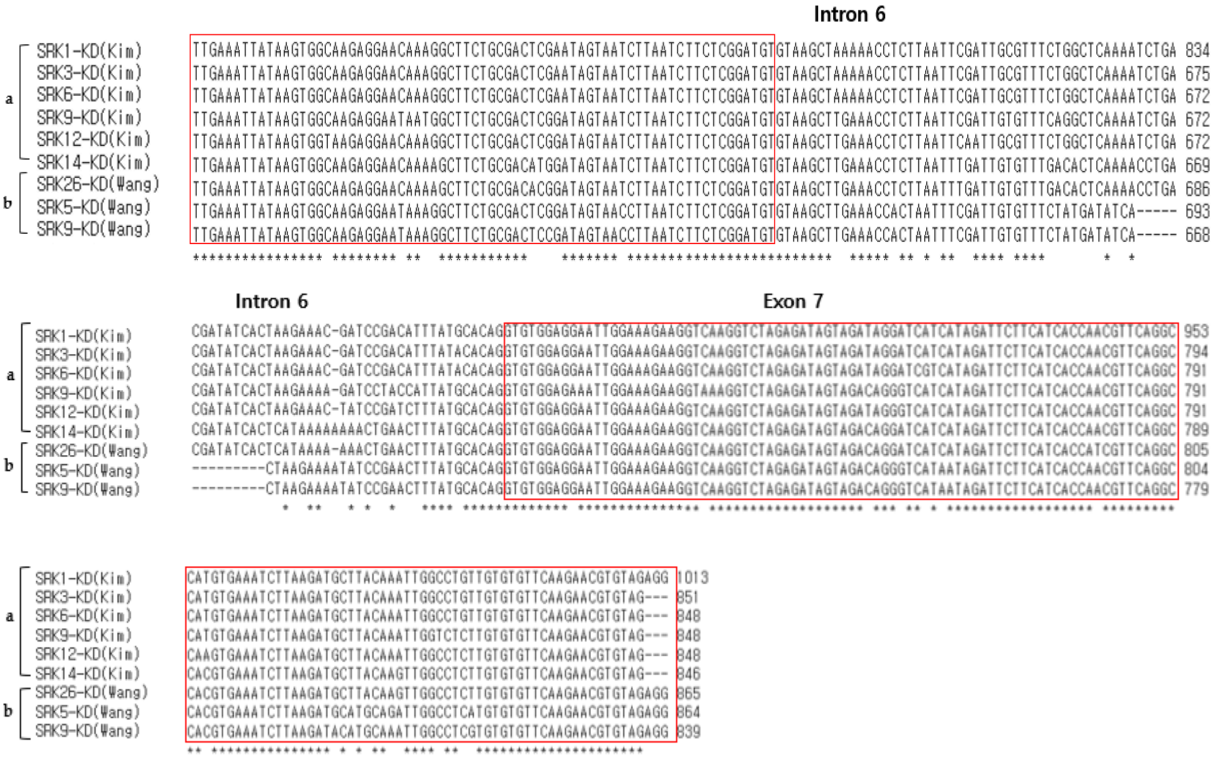

Figure S5. *Continued.*

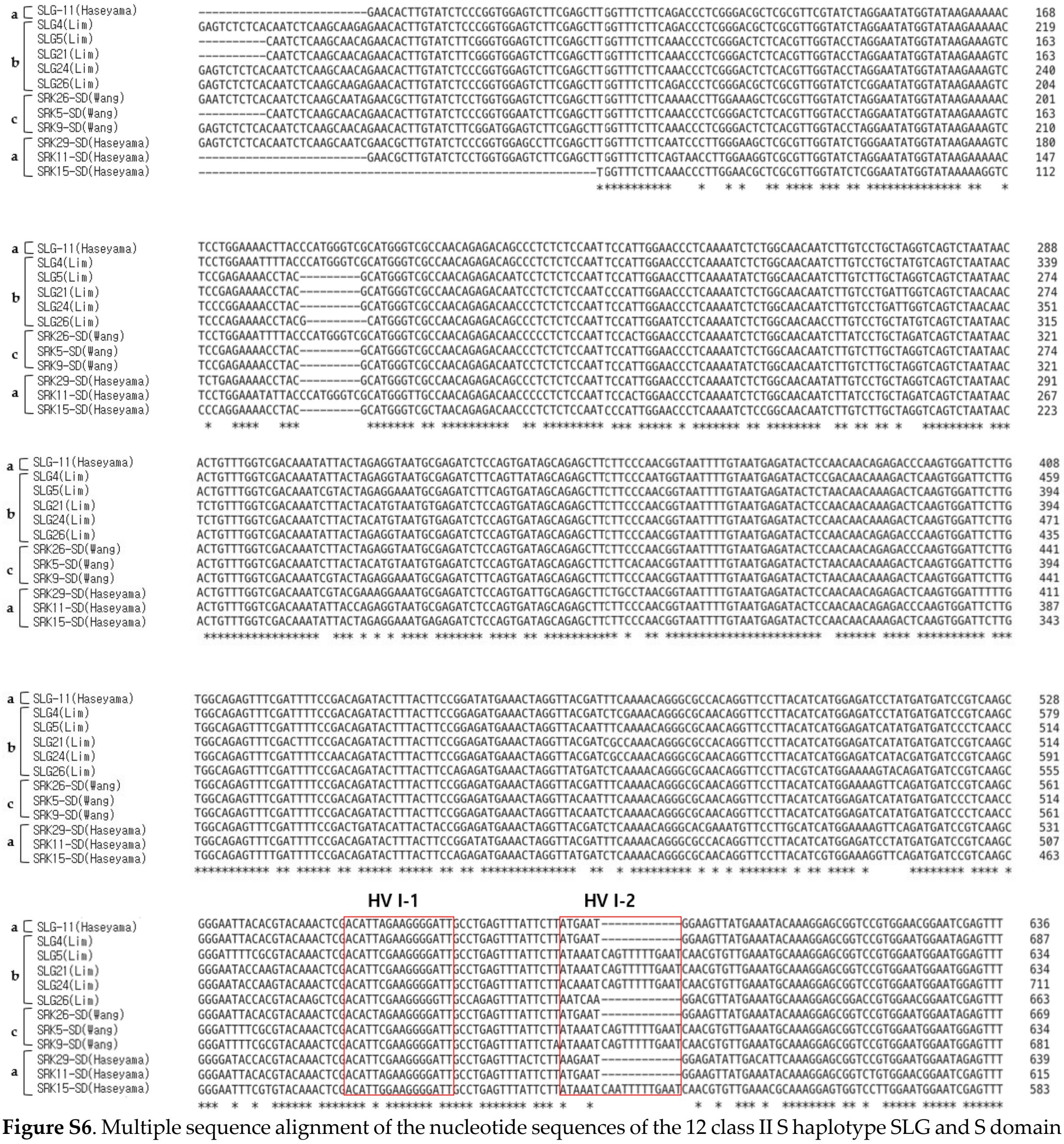

Figure S6. Multiple sequence alignment of the nucleotide sequences of the 12 class II S haplotype SLG and S domain of SRK alleles; a: S haplotype published by Haseyama (2018); b: S haplotype published by Lim (2002); c: S haplotype published by Wang (2018); Red box: hypervariable region I to III of SLG; Asterisk: conserved nucleotide.

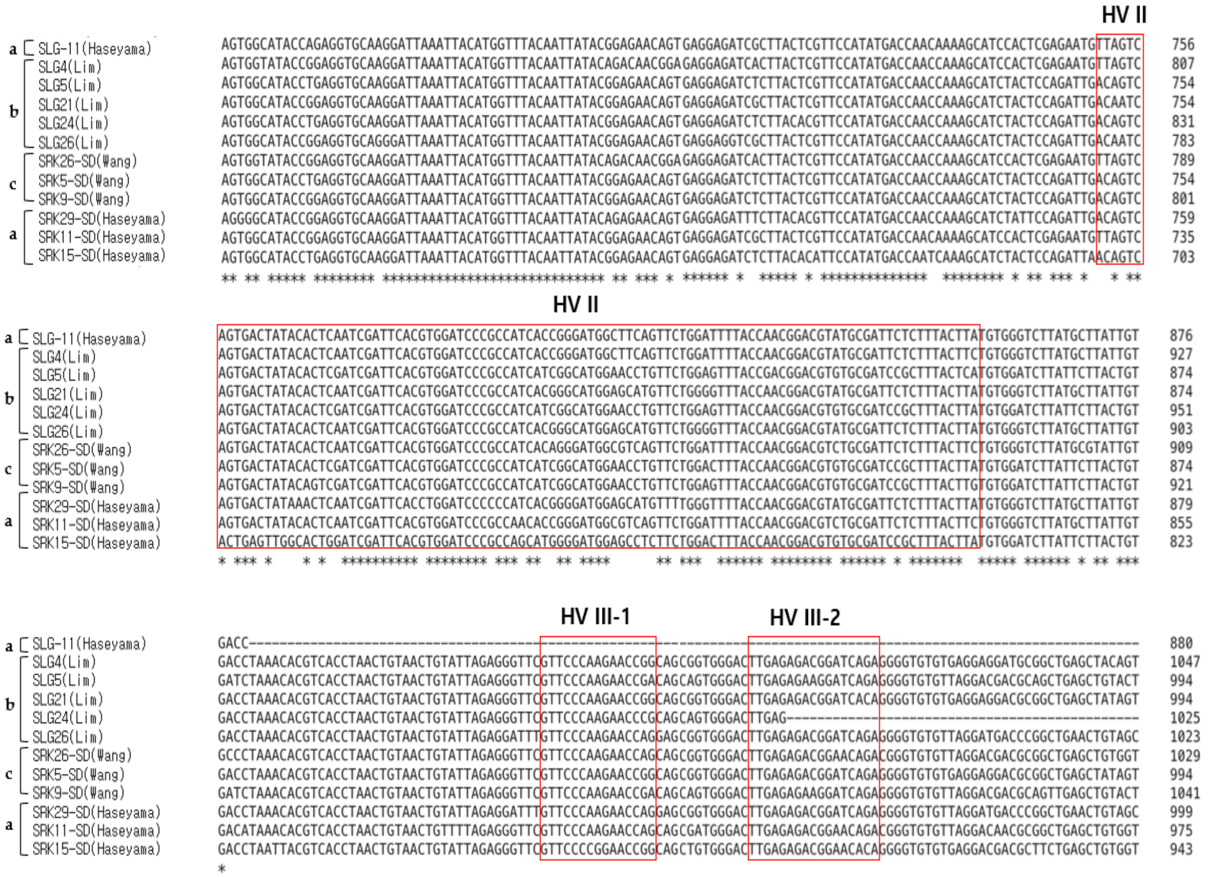

Figure S6. *Continued.*

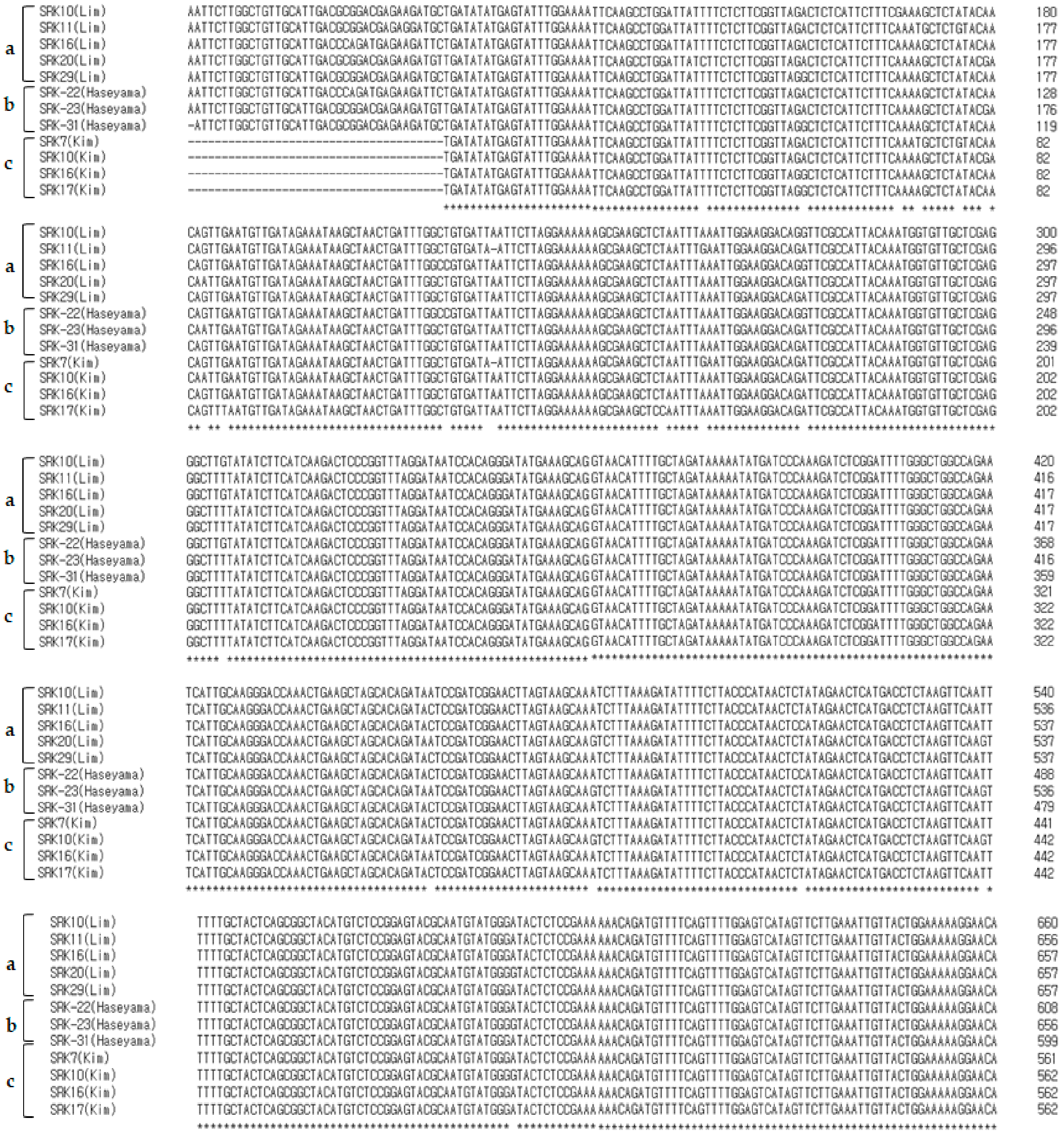

Figure S7. Multiple sequence alignment of SRK10 (Lim, AY052579), SRK16 (Lim, AY052579) and other 10 S haplotypes; Asterisk: conserved nucleotide; a: S haplotype published by Lim (2002); b: S haplotype published by Haseyama (2018); c: S haplotype published by Kim (2016).

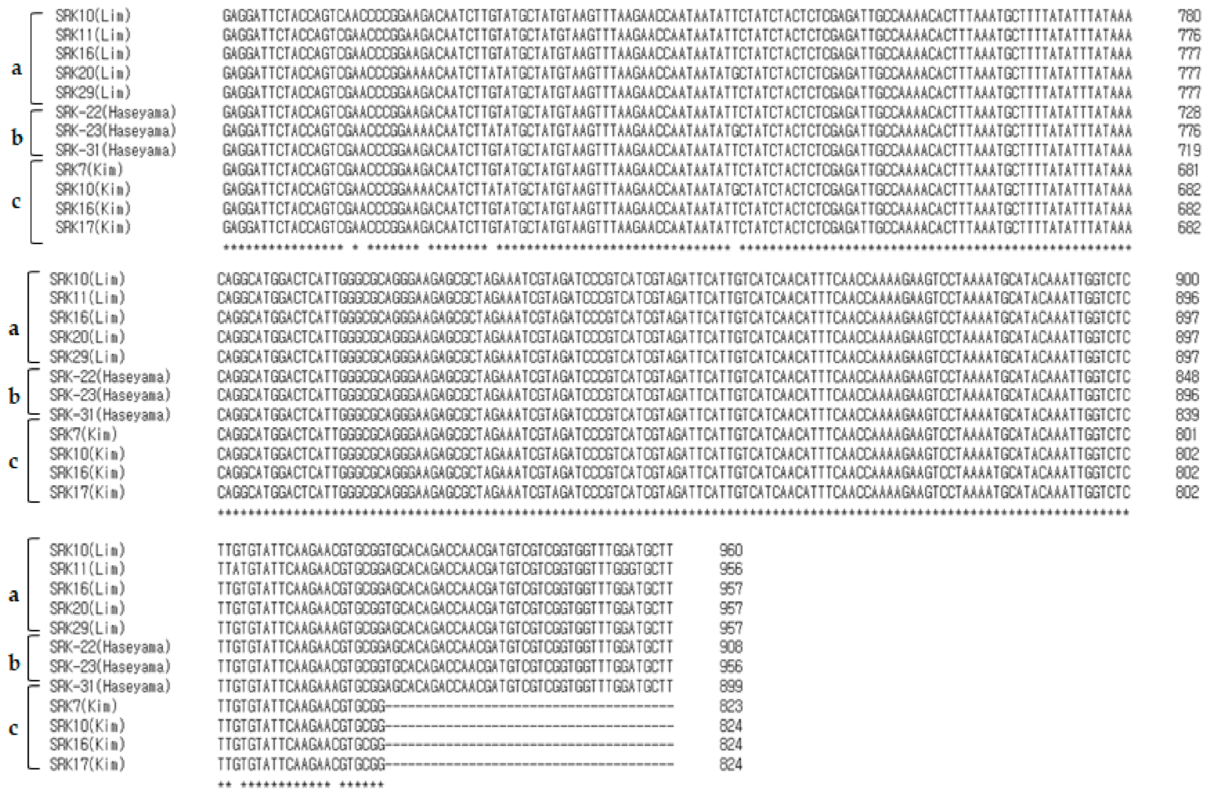

Figure S7. *Continued.*
