# Supplementary material for: Development of S Haplotype-Specific Markers to Identify Genotypes of Self-Incompatibility in Radish (Raphanus sativus L.)
Source: Plants (Basel). 2024 Mar 4;13(5):725. doi: 10.3390/plants13050725 (PMC10934505; doi:10.3390/plants13050725)
Supplement: Supplementary file 1 [file plants-13-00725-s001.zip › Supplementary Table.pdf]

Table S1. Information of class I, II S haplotype universal primer used in this study.

Gene	Primer	Primer sequence (5′ to 3′)	Reference
Class I SRK-KD	UVSRK-F	TGATGAGTTTATGAATGAGGTGA	Delorme et al., 1995
	UVSRK-R	GCTTTCATATTACCGGGCATCGATGA	Delorme et al., 1995
Class II SRK-KD	KS2	GAGGGCGAGAAGATCTTAATT	Seo et al., 2004
	KA2	AAGACKATCATATTACCGAGC	Seo et al., 2004
Class I SLG	SLG-I-F	ATGAAAGGCGTAAGAAAAACCTA	Lim et al., 2002
	SLG-I-R	CCGTGTTTTATTTTAAGAGAAAGAGCT	Lim et al., 2002
	PS22	ATCGATGGGATGAAAAAGTCATCG	Sakamoto et al., 1998
Class II SLG	SLG-II-F	ATGAAAGGGGTACAGAACAT	Chen and Narsrallah, 1990
	SLG-II-R	CTCAAGTCCCACTGCTGCGG	Chen and Narsrallah, 1990
	PS21	GTCAAGTCCCACTGCTGCGGG	Nishio et al., 1996
	Rs9 SLG-F	TCTTCCTTGTCTTGGTTCTATTTC	Cho and Kim, 2021
	Rs9 SLG-R	ACCCCAGAACATGCTCCAT	Cho and Kim, 2021
	UVSLGII-F	TCATCCTGCCCTTTCGATCTATG	Designed in this study
	UVSLGII-R	AAAACACACAACCCAATCCGCCA	Designed in this study

Table S2. Information of 9 S haplotype-specific primer developed in this study.

Gene	Primer	Primer sequence(5′ to 3′)	GC(%)	Tm(℃)
S1 (Lim)	KD1-F	TTGCAAGGGATGAGACGGAA	50%	55.4℃
	KD1-R	CCAACTGCTTACTTGCTGTTTCAT	41.67%	56.7℃
	SLG1-F	CCCGAAGCTTTCCTGAGTTT	50%	55.4℃
	SLG1-R	TTTTCCATATTCCTGGGATC	40%	51.3℃
S4 (Lim)	SLG4-F	TTTTACCCATGGGTCGCAT	47.37%	53.2℃
	SLG4-R	TGATCTCCTCTCCGTTGTCT	50%	55.4℃
S5 (Lim)	SLG5-F	CAATTTCAAAACAGGGCGCAACAGG	48%	60.6℃
	SLG5-R	TCCACATGAGTAAAGCGGATCG	50%	58.4℃
S8 (Lim)	KD8-F	TACCAACATCAGTATCCCATAT	36.36%	52.7℃
	KD8-R	AATGCATTTAAAGTGTTTGAGCAA	29.17%	51.8℃
	SLG8-F	GAGACGCAAGTGGATTATTGT	42.86%	54.1℃
	SLG8-R	ATAAGCGTCATTATCCTCTGTGA	39.13%	54.9℃
S10 (Lim)	KD10-F	AGAGGATTCTACCAGTCAACCC	50%	58.4℃
	KD10-R	GGATTCATCGTGTCGGATGGC	57.14%	60.3℃
	SLG10-F	CTACCTATTGCAAAGCGGCG	55%	57.4℃
	SLG10-R	CGCTCTTCATTCGAGCGATT	50%	55.4℃
S16 (Lim)	SLG16-F	GAATGAGTACCTCAGGGTATT	42.86%	54.1℃
	SLG16-R	CTAACCCGTCTCCCAGATC	57.89%	57.3℃
S18 (Lim)	KD18-F	ACGAAACGGAAGCTAGCACA	50%	55.4℃
	KD18-R	GTAGAGTTATGGGTTCTCTTTG	40.91%	54.3℃
	SLG18-F	ACTTTCCAGTGCATCGGGTT	50%	55.4℃
	SLG18-R	CTGCTGCACGTTCCAGGGAT	60%	59.5℃
S21 (Lim)	SLG21-F	GTTACGATCGCCAAACAGGGCGCC	62%	65.3℃
	SLG21-R	CTCCTCACACACCCCTGTGATCCG	62%	65.3℃
S26 (Lim)	SLG26-F	CTTAATCAAGGACGTTATGAAATG	33.33%	53.4℃
	SLG26-R	TAAGCGACCTCCTCACTGTTC	52.38%	58.2℃

Table S3. Result of BLASTN 2.14.1+ using SLG21(Lim) nucleotide sequence to *Raphanus sativus* genome as sembly 'QZ-16' (GCA_902824885.1). SLG21(Lim) sequence is obtained from 'SJ-21' radish breeding line; Bo ld letters: putative region of SLG21(Lim) and SRK21(Lim) S domain.

Query	Subject (Acc.)	Iden (Region)	Length	Mismatch	E value	Bit score
SLG21 (Lim) (1,055bp)	**chromosome: 8 (LR778317.1)**	**100% (23369737~23370791)**	**1055**	**0**	**0.0**	**1949**
	**chromosome: 8 (LR778317.1)**	**94.218% (23333840~23334894)**	**1055**	**61**	**0.0**	**1611**
	chromosome: 8 (LR778317.1)	75.373% (23031621~23032018)	392	85	5.91e-44	182
	chromosome: 2 (LR778311.1)	81.144% (42830387~42831429)	1055	167	0.0	824
	chromosome: 2 (LR778311.1)	76.93% (9982886~9981803)	1055	168	551	1055
	chromosome: 1 (LR778310.1)	77.332% (10017398~10016505)	1055	150	8.65e-137	490
	chromosome: 1 (LR778310.1)	85.876% (10029198~10029022)	169	16	2.13e-43	180
